# Study on Mechanism of Yiqi Yangyin Jiedu Recipe Inhibiting Triple Negative Breast Cancer Growth: A Network Pharmacology and *In Vitro* Verification

**DOI:** 10.1155/2022/9465124

**Published:** 2022-03-27

**Authors:** Xin Sun, Panling Xu, Fengli Zhang, Ting Sun, Haili Jiang, Mei Zhang, Ping Li

**Affiliations:** ^1^Anhui Provincial Hospital, Cheeloo College of Medicine, Shandong University, Jinan, Shandong 250012, China; ^2^Anhui Provincial Hospital, Hefei, Anhui 230032, China; ^3^Department of Chinese Integrative Medicine Oncology, The First Affiliated Hospital of Anhui Medical University, Hefei, Anhui 230022, China

## Abstract

**Background:**

The present study explores the potential mechanism of Yiqi yangyin jiedu Recipe (YQYYJDR) on triple negative breast cancer via adopting network pharmacology and experimental validation.

**Materials and Methods:**

The potential active compounds and target genes of YQYYJDR were screened out from TCMSP database with OB ≥ 30% and DL index ≥ 0.18. The potential pathways and function enrichment were identified from Metascape website. MDA-MB-231 and MDA-MB-468 cells were tested for cell viability, invasion, and apoptosis by *in vitro* and *in vivo* experiments.

**Results:**

A total of 153 bioactive compounds and 281 target genes of YQYYJDR were retrieved from TCMSP database. The top 5 enrichment pathways of YQYYJDR target genes include pathways in cancer, AGE-RAGE signaling pathway in diabetic complications, proteoglycans in cancer, IL-17 signaling pathway, and platinum drug resistance. 65 target genes were included in the pathway of cancer. Biological function enrichment analysis of 65 genes showed YQYYJDR inhibited tumor growth mainly through apoptotic pathway. *In vitro* experiments showed that YQYYJDR could inhibit the proliferation and invasion of MDA-MB-231 and MDA-MB-468 cells, arrest cells in S stage, and induce cell apoptosis. YQYYJDR upregulated BAX, caspase3, and cleaved caspase3 expression and downregulated BCL2 expression. *In vivo* experiments showed that YQYYJDR could inhibit tumor growth.

**Conclusions:**

In this study, network pharmacology and experiment were used to explore the mechanism of YQYYJDR on triple negative breast cancer. *In vitro* and *in vivo* experiments showed that YQYYJDR could inhibit the growth of triple negative breast cancer and induce cell apoptosis. Apoptosis pathway plays a significant role in the treatment of triple negative breast cancer.

## 1. Introduction

Triple negative breast cancer (TNBC) is an aggressive cancer with poor prognosis, which accounts for less than 30% in breast cancer [1]. It is characterized by negative expression of estrogen receptor (ER), progesterone receptor (PR), and human epidermal growth factor receptor 2 (HER-2) [2]. The recurrence and disease metastasis rates in TNBC are high, and the median survival for patients with advanced disease is about 18 months [3], which emphasizes the importance of developing more effective therapies for patients. The current treatments on TNBC include chemotherapy, targeted therapy, and immunotherapy. However, these treatments do not work well, and patients' quality of life is impaired [4]. Therefore, it is very important to find an alternative treatment for TNBC.

Yiqi yangyin jiedu Recipe (YQYYJDR) is a representative prescription for the treatment on breast cancer. YQYYJDR is composed of Angelica Sinensis, Astragalus Membranaceus, Radix Pseudostellariae, Prepared Radix Rehmanniae, Fructus Amomi, Radix Scrophulariae, Semen Coicis, Fructus Lycii, Rhizoma Atractylodis, Radix Isatidis, Lonicera Japonica, Chrysanthemum, Fructus Forsythiae, Glossy Privet Fruit, Subprostrate Sophora, and Chinese Yam. Researches had proved that YQYYJDR can improve the clinical effect of breast cancer treatment [5]. However, the efficacy and mechanism of YQYYJDR against TNBC still need to be elucidated.

Network pharmacology is an emerging subject for explaining the mechanism of drug acting on disease [6]. The “drug-target-gene” model provides new perspective and methods to explore the potential targets in compound preparations, and furthermore, network pharmacology provides a more efficient platform to reveal the interactions and internal relationships between drugs and diseases [7]. In recent years, network pharmacology has been widely employed in compound preparation mechanism exploration [8, 9]. However, the reports on pharmacology network analysis of YQYYJDR has not been seen yet.

In this study, the active chemical compounds and target genes of YQYYJDR were searched using network pharmacology methods. And the drug-gene-disease network and function pathways were analyzed. *In vitro* and *in vivo* experiments were used to validate the antitumor effect and potential targets of YQYYJDR treating on TNBC.

## 2. Material and Methods

### 2.1. YQYYJDR-Related Compounds and Potential Targets

Active compounds and potential targets of YQYYJDR were searched in Traditional Chinese Medicine Database and Analysis Platform (TCMSP, https://tcmsp-e.com/), with the oral bioavailability (OB) ≥ 30% and drug − likeness (DL) ≥ 0.18. OB represents the percentage of drugs that reach the systemic circulation at the same oral dose; DL is used to assess the degree to which a desired compound is “drug-like,” which helps to optimize drug pharmacokinetics and drug properties such as solubility and chemical stability [10]. Compounds that meet the above criteria will be considered as bioactive compounds. Then, the potential targets screened from TCMSP were imported into UniProt (http://www.uniprot.org/) to search their information, including gene name, functions, and gene ID [11].

### 2.2. Construction of YQYYJDR Network

The screened active compounds and potential targets were introduced into Cytoscape 3.7.2 (http://www.cytoscape.org/) to draw the activity “herb-compounds-targets” network [12]. The nodes in this network represent the active ingredients and targets of YQYYJDR; the interactions and internal relationships between the active compounds and the targets were encoded by edges.

### 2.3. Potential Pathways of YQYYJDR

The targets of YQYYJDR were inputted into Metascape website (https://metascape.org/) for analyzing Kyoto Encyclopedia of Genes and Genomes (KEGG) pathway and Gene Ontology Consortium (GO) Biological Processes. KEGG and GO analyses were enriched with *p* value < 0.01. Metascape is a comprehensive annotated and analytical resource for experimental biologists [13].

### 2.4. Preparation of YQYYJDR Aqueous Extract

YQYYJDR consisted of 20 g Angelica Sinensis, 30 g Astragalus Membranaceus, 15 g Radix Pseudostellariae, 18 g Prepared Radix Rehmanniae, 6 g Fructus Amomi, 15 g Radix Scrophulariae, 30 g Semen Coicis, 15 g Fructus Lycii, 15 g Rhizoma Atractylodis, 12 g Radix Isatidis, 20 g Lonicera Japonica, 20 g Chrysanthemum, 15 g Fructus Forsythiae, 15 g Glossy Privet Fruit, 15 g Subprostrate Sophora, and 15 g Chinese Yam. All crude herbs were provided by the Department of Pharmacy, The First Affiliated Hospital of Anhui Medical University. 1000 mL water was prepared to boil all above mixed herbs for 2 h, and the herbal extract of YQYYJDR was got. The obtained herbal extract was centrifuged for 30 min at 10000 rpm, then extracted twice, mixed with supernatants, and evaporated to dryness. The herbal powder was redissolved in water at a concentration of 40 mg/mL and then filtered with a 0.22 *μ*m pore-size filter. Finally, the Chinese herb liquors were stored at -20°C for further use.

### 2.5. Cell Culture

The human breast cancer cell lines MDA-MB-231 and MDA-MB-468 were purchased from American Type Culture Collection (ATCC, USA). MDA-MB-231 and MDA-MB-468 cells were maintained in Leibovitz's L-15 medium (Gibco, Massachusetts, USA), which supplemented with 10% fetal bovine serum (FBS, Gibco, Massachusetts, USA), 100 U/mL penicillin, and 100 mg/mL streptomycin (Gibco, Massachusetts, USA). All cells were incubated at 37°C in a humidified incubator with 5% CO2. The morphology of the cell lines was regularly assessed.

### 2.6. Cell Viability Assay

MTT assay was used to detect the cytotoxicity of YQYYJDR on TNBC cell lines. In brief, 100 *μ*L cell suspension (1 × 10^5^ cells/mL) was seeded into 96-well plates; after incubation overnight, several concentrations of YQYYJDR were added into each well followed by a 48 h incubation. 10 *μ*L 3-(4,5-dimethyl-2-thiazolyl)-2,5-d iphenyl-2-H-tetrazolium bromide (MTT, 5 mg/mL; Sigma, USA) in phosphate-buffered saline (PBS, Gibco, USA) was added and incubated at 37°C for 4 h to produce formazan. 100 *μ*L DMSO (Sigma, USA) was added, and the absorbance of the microplate reader was measured at 490 nm (SpectraMax ABS plus, Molecular Devices, USA).

### 2.7. Cell Cycle Analysis

A cell cycle and apoptosis analysis kit was used to test for cell cycle arrest. 5 × 10^5^ cells were inoculated in each well of 6-well plates. After the cells were incubated overnight, they were treated with increasing doses of YQYYJDR for 48 hours. The cells were digested with trypsin to obtain the cell deposits, which were then fixed with precooled 70% ethanol; the cells were stained with propidium iodide solution and then detected by flow cytometry.

### 2.8. Apoptosis Assay

An Annexin V-FITC Apoptosis Detection Kit (Beyotime, Shanghai, China) was used to analyze cell apoptosis level. In brief, MDA-MB-231 and MDA-MB-468 cells (5 × 10^5^ cells/mL) were seeded in 6-well plates, then preincubated for 24 h. Different doses of YQYYJDR were added in 6-well plates and then incubated at 37°C for 48 h. Annexin V-FITC and propidium iodide staining solution were added to incubate for 20 minutes. Then, the cell apoptosis level was evaluated using a flow cytometer (CytoFLEXS, Beckman COULTER, USA).

### 2.9. Transwell Analysis

Cell invasion efficiency was verified by transwell assay. In brief, 200 *μ*L Matrigel matrix was added into each well of a 24-well plate, 2 × 10^5^ cells were cultured DMEM without FBS for 8 h, then inoculated in each well of a 24-well plate, and DMEM with 20% FBS were added into the bottom. After incubation for 24 hours, cells in upper chamber were fixed with 4% paraformaldehyde stained and stained with crystal violet staining solution after wiping the matrix. An optical microscope was used to observe.

### 2.10. RT-PCR

Total RNA from TNBC cells was extracted by using TRIzol reagent (Invitrogen, USA). Then, cDNA were obtained using TaKaRa PrimeScript RT reagent Kit. ABI 7900HT Real-Time PCR system (Applied Biosystems, USA) was used to amplify cDNA for comparing gene expression between different experimental groups. All reactions were run in triplicate. The sequences of the primers for genes are shown in Table 1.

### 2.11. Western Blotting

In order to verify the related pathways of drug action on cells, the cell protein samples were extracted by RIPA lysis buffer. The protein concentration of each protein sample was tested by BCA protein assay kit. SDS-PAGE protein loading buffer was added to protein samples for protein denaturation. After electrophoresis, transfer, and blocking, primary antibody was used to incubating with 0.22 *μ*m PVDF membrane for 1 hour, followed by secondary antibody incubation and detection. The primary antibody in this study was shown below: *α*-Tubulin Rabbit Polyclonal Antibody (AF0001, Beyotime, China), Anti-BAX rabbit polyclonal antibody (D220073, BBI, China), Anti-BCL2 rabbit polyclonal antibody (D160117, BBI, China), and Anti-CASP3 rabbit polyclonal antibody (D320074, BBI, China).

### 2.12. In Vivo Experiment

Twelve BALB/c nude mice were obtained from GemPharmatech Co., Ltd. These mice were kept in the Laboratory Animal Center, Anhui Medical University. After one week of adaptive feeding, 1 × 10^7^ MDA-MB-231 cells were injected under the skin of the right armpit of the mice. 7 days after injection, these mice were randomly divided into 3 groups with 6 mice in each group. One group was intragastric with 0.2 mL normal saline, and the other two groups were intragastric with 20 mg/kg and 40 mg/kg YQYYJDR once a day. 7 days after subcutaneous tumor injection, tumor length and diameter, as well as body weight of mice, were recorded every 3 days. After 21 days drug intervention, pentobarbital sodium was used at 250 mg/kg for mice anesthesia; subcutaneous tumors were broken off. The tumors were fixed in 4% paraformaldehyde for further detection.

### 2.13. Hematoxylin-Eosin Staining and Immunohistochemistry

Tumors fixed with 4% paraformaldehyde were embedded in paraffin, then sliced into 4 *μ*m slices. Hematoxylin and Eosin Staining Kit was used to stain slices for 10 minutes, and then, a light microscope was used for analysis. As for immunohistochemistry, paraffin sections were dewaxed and sealed with goat serum; primary antibodies and second antibodies were incubated with paraffin sections. Tissue is then visualized under a light microscope (200x, Leica, Wetzlar, Germany).

### 2.14. Statistical Analysis

Statistical analysis was performed with the GraphPad Prism 9 software. All data were obtained from three independent experiments; data were analyzed by using Student's *t*-test and described as the mean ± SD. *P* values between groups were less than 0.05, indicating statistically significant differences.

## 3. Results

### 3.1. Identification of Bioactive Compounds and Targets in YQYYJDR

**Figure 1:**
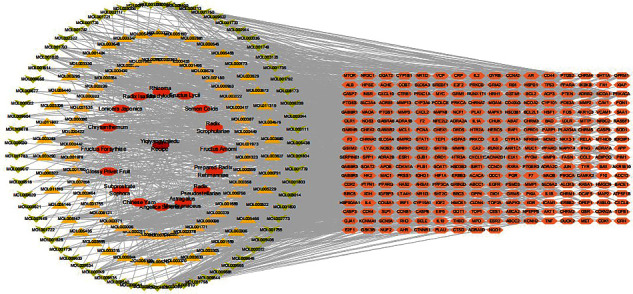
The “herb-compounds-targets” network diagram of YQYYJDR.

With the OB threshold ≥ 30% and DL index ≥ 0.18, a total of 153 bioactive compounds were retrieved from YQYYJDR, 2 of which belong to Angelica Sinensis, 18 of which belong to Astragalus Membranaceus, 6 to Radix Pseudostellariae, 2 to Prepared Radix Rehmanniae, 9 to Fructus Amomi, 5 to Radix Scrophulariae, 6 to Semen Coicis, 36 to Fructus Lycii, 4 to Rhizoma Atractylodis, 35 to Radix Isatidis, 17 to Lonicera Japonica, 18 to Chrysanthemum, 19 to Fructus Forsythiae, 9 to Glossy Privet Fruit, 13 to Subprostrate Sophora, 12 to Chinese Yam. 281 targets were predicted out of the 153 bioactive compounds. A “herb-compounds-targets” network was constructed (Figure 1). The network consists of 450 nodes (153 bioactive compounds and 281 targets). It is worth noting that this network includes some compounds with multiple targets, particularly the compounds quercetin (MOL000098), kaempferol (MOL000422), beta-sitosterol (MOL000358), wogonin (MOL000173), and 7-O-methylisomucronulatol (MOL000378) with degree ≥ 45.

### 3.2. Potential Pathways of YQYYJDR

**Figure 2:**
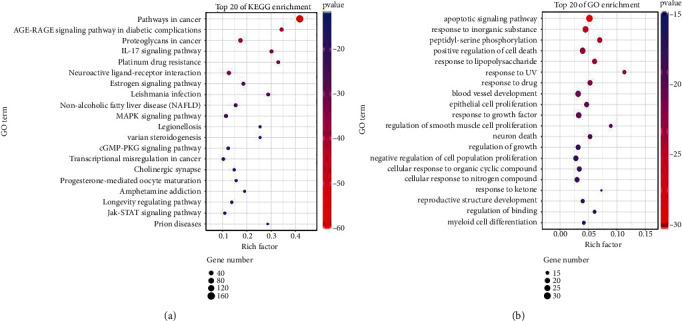
The KEGG pathway and GO Biological Processes of YQYYJDR.

KEGG pathway enrichment analysis was performed in Metascape website. In total, forty pathways were observed to be significantly associated with the gene input group (*P* < 0.001); the top 20 pathways are shown in Figure 2(a). The main pathways included pathways in cancer, AGE-RAGE signaling pathway in diabetic complications, proteoglycans in cancer, IL-17 signaling pathway, and platinum drug resistance. 65 target genes were included in the pathway of cancer. Biological function enrichment analysis of 65 genes showed YQYYJDR inhibited tumor growth mainly through the apoptotic pathway, shown as Figure 2(b).

### 3.3. Apoptosis Network of YQYYJDR

**Figure 3:**
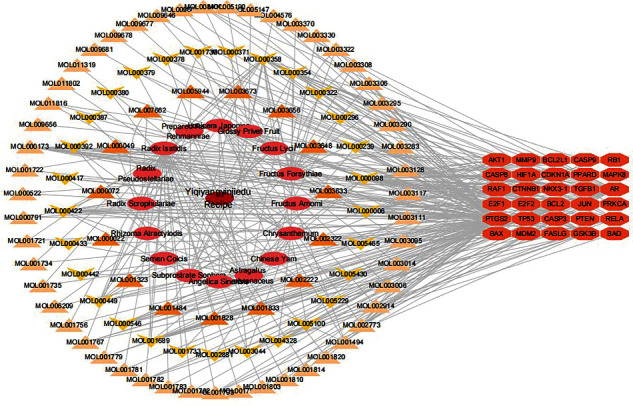
The apoptosis genes/compounds/YQYYJDR network.

A total of 30 apoptosis genes in YQYYJDR were obtained. The apoptosis genes/compounds/YQYYJDR network was drawn by Cytoscape 3.7.2, as shown in Figure 3. It can be seen that the main compounds of YQYYJDR are quercetin (MOL000098), beta-sitosterol (MOL000358), luteolin (MOL000006), kaempferol (MOL000422), and acacetin (MOL001689).

### 3.4. YQYYJDR Suppressed Cell Growth and Induced Cell Apoptosis In Vitro

**Figure 4:**
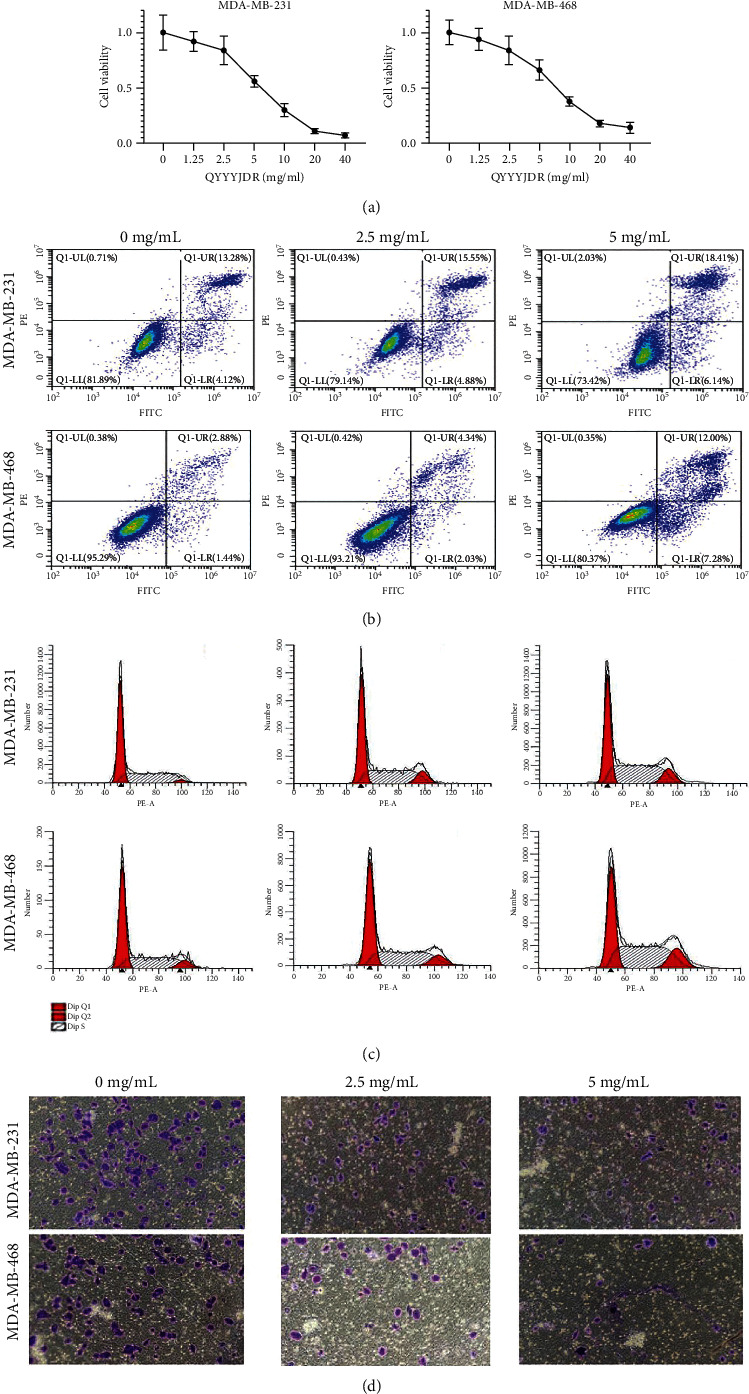
YQYYJDR suppressed in *vitro* triple negative breast cancer cell growth. (a) YQYYJDR showed a dose-dependent effect on the viability of breast cancer cells. (b) YQYYJDR induced MDA-MB-231 and MDA-MB-468 cell apoptosis. (c) YQYYJDR blocked MDA-MB-231 and MDA-MB-468 cells in the S phase. (d) YQYYJDR inhibited MDA-MB-231 and MDA-MB-468 cell invasion.

To verify the efficacy of YQYYJDR on TNBC cancer cells, the cell viability assay of MDA-MB-231 and MDA-MB-468 cells was performed. YQYYJDR showed a dose-dependent effect on the viability of cancer cells in increasing concentrations (0-40 mg/mL) at 48 h. The IC_50_ value at 48 h was 6.095 mg/mL for MDA-MB-231 cells and 7.877 mg/mL for MDA-MB-468 cells, as shown in Figure 4(a). Based on the observed IC_50_ values, YQYYJDR was used at concentrations of 0, 2.5, and 5 mg/mL in subsequent analysis. FITC/PE staining was used to evaluate cell apoptosis in cancer cells treated with YQYYJDR. YQYYJDR could induce cancer cell apoptosis, as shown in Figure 4(b). YQYYJDR arrested MDA-MB-231 and MDA-MB-468 cells in the S phase and inhibited cell invasion, as shown in Figures 4(c) and 4(d).

### 3.5. Apoptotic Mechanism Plays a Significant Role in the Inhibition of YQYYJDR on TNBC

**Figure 5:**
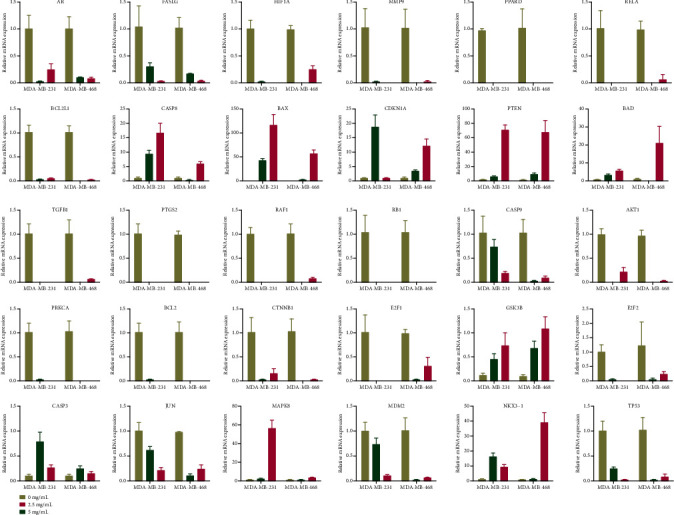
The mRNA expression of apoptosis-related genes after the treatment of YQYYJDR. The mRNA expressions of CASP8, BAX, CDKN1A, PTEN, BAD, CASP3, MAPK8, GSK3B, and NKX3-1 were upregulated in cancer cells, and the mRNA expressions of AR, FASLG, HIF1A, MMP9, PPARD, RELA, BCL2L1, TGFB1, PTGS2, RAF1, RB1, CASP9, AKT1, PRKCA, BCL2, CTNNB1, E2F1, E2F2, JUN, MDM2, and TP53 were downregulated in cancer cells after the treatment with YQYYJDR.

**Figure 6:**
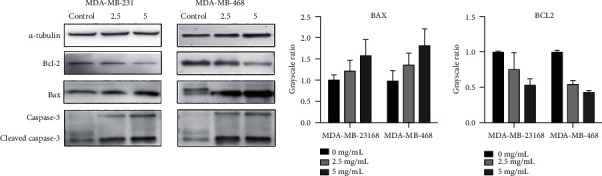
Protein expression in MDA-MB-231 and MDA-MB-468 cells after YQYYJDR intervention. YQYYJDR upregulated BAX, caspase3, and cleaved caspase3 and downregulated BCL2 expression.

These apoptotic genes were validated by RT-PCR *in vitro*, as shown in Figure 5; the mRNA expressions of CASP8, BAX, CDKN1A, PTEN, BAD, CASP3, MAPK8, GSK3B, and NKX3-1 were upregulated in cancer cells, and the mRNA expressions of AR, FASLG, HIF1A, MMP9, PPARD, RELA, BCL2L1, TGFB1, PTGS2, RAF1, RB1, CASP9, AKT1, PRKCA, BCL2, CTNNB1, E2F1, E2F2, JUN, MDM2, and TP53 were downregulated in cancer cells after the treatment with YQYYJDR. The results showed that YQYYJDR could induce cell apoptosis. Apoptosis-related proteins, such as BAX, BCL2, caspase3, and cleaved caspase3, were validated by western blotting *in vitro*; the results showed that YQYYJDR upregulated BAX, caspase3, and cleaved caspase3 expression and downregulated BCL2 expression, as shown in Figure 6.

### 3.6. YQYYJDR Inhibited TNBC Tumor Growth In Vivo

**Figure 7:**
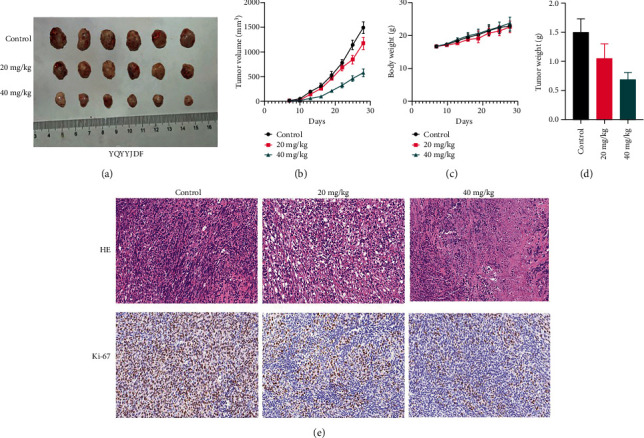
YQYYJDR inhibited tumor growth in mice. (a, b, d) YQYYJDR inhibited MDA-MB-231 mouse xenograft tumor growth. (c) YQYYJDR had no effect on mouse weight. (e) YQYYJDR inhibited Ki-67 expression in tumor tissues.

MDA-MB-231 mouse xenograft tumors were used to evaluate the tumor suppressive effect of YQYYJDR. The result showed that YQYYJDR at 40 mg/kg inhibited tumor growth significantly with 53% inhibition rate (*P* < 0.01). And YQYYJDR had no toxic effect on the body weight of the mice. HE and immunohistochemistry results showed that YQYYJDR inhibited the expression of Ki-67 protein in tumors, as shown in Figure 7.

## 4. Discussion

YQYYJDR has been used in clinic for breast cancer treatment [5]. Its main compounds include quercetin, kaempferol, beta-sitosterol, wogonin, and 7-O-methylisomucronulatol. Quercetin and wogonin have been reported to suppress cell proliferation and metastasis and induce cell apoptosis in TNBC cancer cells [14–17]. Kaempferol can suppress proliferation and induce apoptosis and autophagy in human lung cancer cells [18]. beta-Sitosterol could induce G1 arrest and cause depolarization of mitochondrial membrane potential and sensitize cells to TRAIL-induced apoptosis in breast carcinoma MDA-MB-231 cells [19, 20]. However, the mechanism of YQYYJDR on TNBC has not been reported. A variety of compounds in YQYYJDR have anticancer effect, which suggests that the compound preparation of various herbs will receive certain attention in the future research.

The active compounds and target genes of traditional herbs have been widely explored by using network pharmacology methods, and it provides a new thought in the research of traditional medicine [21]. However, some new problems are also emerging, such as mixed research, decentralized and irregular data, and lack of scientific validation [22]. Recently, the Network Pharmacology Evaluation Method Guidance has been issued to solve these new problems [22]. In this study, the data of traditional herbs were extracted from TCMSP database, and the KEGG pathways were found out in Metascape database and proofed by experiments, thus exploring the mechanism of YQYYJDR on TNBC. As the information of compound targets in TCMSP database is collected from existing research data, some medicinal materials with little research may not be comprehensive in the database, which leads to certain selection bias in the prediction of compound preparation targets. However, this problem will be improved with the perfection of database information.

The network pharmacology results of YQYYJDR show that apoptotic mechanism may play a significant role in the inhibitory effects of YQYYJDR on TNBC. Main compounds that exert apoptosis-inducing functions include quercetin, beta-sitosterol, luteolin, kaempferol, and acacetin. The *in vitro* experiment showed that YQYYJDR could inhibit MDA-MB-231 and MDA-MB-468 cell proliferation and invasion, induce cells apoptosis, arrest cells in S stage, and regulate mRNA expression. The upregulation RNAs include CASP8, BAX, CDKN1A, PTEN, BAD, CASP3, MAPK8, GSK3B, and NKX3-1, and the mRNA expressions of AR, FASLG, HIF1A, MMP9, PPARD, RELA, BCL2L1, TGFB1, PTGS2, RAF1, RB1, CASP9, AKT1, PRKCA, BCL2, CTNNB1, E2F1, E2F2, JUN, MDM2, and TP53 were downregulated. The result of western blotting showed that YQYYJDR can induce cell apoptosis by regulating the expression of BAX, BCL2, and caspase3. Combined with above results, YQYYJDR induced apoptosis of triple negative breast cancer cells by regulating apoptosis-related proteins, thus playing a role in inhibiting tumor growth.

Key compounds that regulate apoptosis in YQYYJDR include quercetin, beta-sitosterol, and luteolin. Quercetin had been reported to potentiate the antimetastatic effect of 5-fluorouracil and docetaxel on the MDA-MB-231 cell line through induction of apoptosis and modulation of PI3K/AKT, MAPK/ERK, and JAK/STAT3 signaling pathways [23, 24]. Additionally, quercetin increased abundance of the proapoptotic protein Bax and decreased the levels of antiapoptotic protein Bcl-2 [25]. There are few such studies about the mechanism beta-sitosterol on MDA-MB-231; only few articles had shown that it induces cell apoptosis [20, 26]. Luteolin could enhance paclitaxel-induced apoptosis in human breast cancer MDA-MB-231 cells by blocking STAT3 [27]. Most of the existing studies focus on the mechanism of TCM monomers enhancing the efficacy of chemotherapy for breast cancer, partly because of the poor tumor suppressive effect of TCM monomers in mice, and on the other hand, TCM monomers are in the early stage of basic research and cannot be applied in clinical practice. As compound preparations of these monomers, YQYYJDR has been widely used in clinical practice, so their clinical significance is greater than that of traditional Chinese medicine monomers. Combined with the *in vivo* results in our study, we believe that the compound preparation has better tumor inhibition effect.

There are several limitations to this study. First, molecular docking between drugs and target proteins was not used to predict possible drug targets in this study; second, the target proteins of apoptosis were not knocked out or overexpression to determine whether apoptotic targets are the main targets of drug action; third, animal experiments were used in this study to verify the efficacy of YQYYJDR; however, the mechanism of action *in vivo* remains unclear; fourth, the HPLC result and pharmacokinetics of compound preparations have not been studied *in vivo*. Therefore, it is necessary to further explore the effect of YQYYJDR on TNBC and clarify its mechanism.

## 5. Conclusions

In this study, network pharmacology techniques and experimental methods were used to explore the mechanism on triple negative breast cancer with YQYYJDR. *In vitro* and *in vivo* experiments showed that YQYYJDR could inhibit the growth of triple negative breast cancer and induce cell apoptosis. Apoptosis pathway plays a significant role in the treatment of triple negative breast cancer.

## Figures and Tables

**Table 1:** The sequences of the primers for genes.

Gene	Forward	Reverse
AKT1	TGACCATGAACGAGTTTGAGTA	GAGGATCTTCATGGCGTAGTAG
AR	CTACATCAAGGAACTCGATCGT	CATGTGTGACTTGATTAGCAGG
BAD	ATGTTCCAGATCCCAGAGTTTG	ATGATGGCTGCTGCTGGTT
BAX	CGAACTGGACAGTAACATGGAG	CAGTTTGCTGGCAAAGTAGAAA
BCL2	GACTTCGCCGAGATGTCCAG	GAACTCAAAGAAGGCCACAATC
BCL2L1	GCATATCAGAGCTTTGAACAGG	GAAGGAGAAAAAGGCCACAATG
CASP3	CCAAAGATCATACATGGAAGCG	CTGAATGTTTCCCTGAGGTTTG
CASP8	CAAACTTCACAGCATTAGGGAC	ATGTTACTGTGGTCCATGAGTT
CASP9	GGAACTCTTCTGCTGCCACTTCTG	GCCCAGGTCTCCAACACAAACAG
CDKN1A	GATGGAACTTCGACTTTGTCAC	GTCCACATGGTCTTCCTCTG
CTNNB1	TGGATTGATTCGAAATCTTGCC	GAACAAGCAACTGAACTAGTCG
E2F1	ATAGTGTCACCACCACCATCAT	GAAAGGCTGATGAACTCCTCAG
E2F2	GAAAGGTCTTGCTGCCCACACTC	GTGATACTGCTGCTGCTGGTCTG
FASLG	CACAGCATCATCTTTGGAGAAG	GTACAGCCCAGTTTCATTGATC
GSK3B	AGGAGAACCCAATGTTTCGTAT	ATCCCCTGGAAATATTGGTTGT
HIF1A	AGTAATGGGATGGCTGGGTCAAATG	GTGCTGGAGAGGATGTGGAGAAAC
JUN	CAAACCTCAGCAACTTCAACC	CTGGGACTCCATGTCGATG
MAPK8	ACACCACAGAAATCCCTAGAAG	CACAGCATCTGATAGAGAAGGT
MDM2	CTTCTAGGAGATTTGTTTGGCG	ATGTACCTGAGTCCGATGATTC
MMP9	CAGTACCGAGAGAAAGCCTATT	CAGGATGTCATAGGTCACGTAG
NKX3-1	GGAAGTTCAGCCATCAGAAGTA	TCGCTTAGTCTTATAGCGTCTG
PPARD	GATCCACGACATCGAGACATT	CGCCATACTTGAGAAGGGTAA
PRKCA	GGTGAAGGACCACAAATTCATC	CACCCGGACAAGAAAAAGTAAC
PTEN	GACCAGAGACAAAAAGGGAGTA	ACAAACTGAGGATTGCAAGTTC
PTGS2	TGTCAAAACCGAGGTGTATGTA	AACGTTCCAAAATCCCTTGAAG
RAF1	TAAGACAAGCAACACTATCCGT	CAGTATTCCAATCTAAGCGTGC
TGFB1	CTGTACATTGACTTCCGCAAG	TGTCCAGGCTCCAAATGTAG
TP53	TTCCTGAAAACAACGTTCTGTC	AACCATTGTTCAATATCGTCCG
RELA	GCAGAGAAGTGGAGTGTCAGGTAAC	GCAGTGTGGGTCAGTGTGTCTAAC
RB1	ATACACGCACAGATACGCTCCTTTC	GGTTAGTGACGCCAGTGACTTCAG
GAPDH	GGAGTGAGTGGAAGACAGAATGGAAG	CCTACAGCAGAGAAGCAGACAGTTATG

## Data Availability

The data used to support the findings of this study are included within the article.
